# Characterization of Full Genome of Rat Hepatitis E Virus Strain from Vietnam

**DOI:** 10.3201/eid1901.121007

**Published:** 2013-01

**Authors:** Tian-Cheng Li, Yasushi Ami, Yuriko Suzaki, Shumpei P. Yasuda, Kumiko Yoshimatsu, Jiro Arikawa, Naokazu Takeda, Wakita Takaji

**Affiliations:** Author affiliations: National Institute of Infectious Diseases, Tokyo, Japan (T.-C. Li, Y. Ami, Y. Suzaki, W. Takaji);; Graduate School of Medicine, Hokkaido University, Sapporo, Japan (S.P. Yasuda, K. Yoshimatsu, J. Arikawa);; Osaka University, Osaka, Japan (N. Takeda)

**Keywords:** hepatitis E virus, rat HEV, rat, genotype, viruses

## Abstract

We amplified the complete genome of the rat hepatitis E virus (HEV) Vietnam strain (V-105) and analyzed the nucleotide and amino acid sequences. The entire genome of V-105 shared only 76.8%–76.9% nucleotide sequence identities with rat HEV strains from Germany, which suggests that V-105 is a new genotype of rat HEV.

Hepatitis E virus (HEV) is a positive-sense single-stranded RNA virus (*1*), classified as the sole member of the genus *Hepevirus* in the family *Hepeviridae* (*2*). Hepatitis E, caused by HEV infection, is a serious public health concern in developing countries and is recognized as sporadic and endemic acute hepatitis (*3*). To date, at least 4 genotypes of HEV have been isolated from humans (*4*). In addition, HEV has been isolated from other mammals, including pigs, wild boars, wild deer, rabbits, ferrets, bats, chickens, and wild rats (*5*–*9*). Much direct evidence indicates that HEV is transmitted from pigs or wild boars to humans, and therefore hepatitis E caused by genotypes 3 and 4 is recognized as a zoonotic disease (*6*,*8*,*10*).

Rat HEV was first isolated from Norway rats in Germany (*7*,*11*). Since then, rat HEV strains have been isolated from wild rats in other areas of Germany and detected in wild rats in the United States and Vietnam (*12*–*14*). Those results suggest that rat HEV infection is not restricted to Germany but is broadly distributed in wild rats throughout the world. The nucleotide sequences of the rat HEV isolated in Germany and the United States are similar; however, the partial sequences of the Vietnam rat HEV strain (V-105, JN040433) have been found to have 78.18%–79.43% identities with isolates from Germany, R63 and R68 (*14*). To confirm whether new genotypes of rat HEV exist, we amplified the entire genome of the rat HEV V-105 strain and analyzed the sequences. We confirmed that the rat HEV strain isolated in Vietnam belongs to a new genotype of rat HEV.

## The Study

The rat HEV used in this study was isolated from a 10% lung homogenate of a wild rat from Vietnam, which was positive for rat HEV RNA by reverse transcription PCR (RT-PCR) (*14*). Because of the limited availability of rat specimens that are positive for HEV RNA, we first transmitted the rat HEV to a laboratory rat (Wistar) to produce a large amount of virus for RNA extraction and genome amplification. After intravenous inoculation of the rat, fecal specimens positive for HEV RNA were collected, and a 10% suspension was prepared as described (*15*). RT-PCR was performed by using Superscript II RNase H^–^ (Invitrogen, Carlsbad, CA, USA) and primer TX30SXN (*14*). The full-length genome of the V-105 strain was amplified by RT-PCR with primers based on the nucleotide sequences of GU345042 and JN040433 (Table 1). All PCR products were purified by using the QIAquick PCR Purification Kit (QIAGEN, Valencia, CA, USA) and cloned into TA cloning vector pCR2.1 (Invitrogen). The nucleotide sequencing was carried out by using an ABI 3130 Genetic Analyzer automated sequencer (Applied Biosystems, Foster City, CA, USA).

**Table 1:** Oligonucleotides used in amplifying the complete genome of the rat HEV Vietnam strain, V-105*

Primers	Product length, bp
Forward ORF1-F1 (1-21)† 5'-GCAACCCCCGATGGAGACCCA-3'‡	
Reverse ORF1-R12 (4149-4171) 5'-GGCGGCCTCGAACTTCTCCTGAA-3'	§
Forward ORF1-F2 (11-30) 5'-ATGGAGACCCATCAGTATGT-3'†	
Reverse ORF1-R1 (431-450) 5'-GTGCAAAAGGAAAGATCAGT-3'	440
Forward ORF1-F9 (388-408) 5'-AGCTAACAACATCCGCCGTTG-3'	
Reverse ORF1-R10 (2197-2217) 5'-TGGGTTCGGTCGAAGGCCTCT-3'†	1,830
Forward ORF1-F16 (2080-2100) 5'-TGCAGCCGTTTATGAGGGAGA-3'	
Reverse ORF1-R16 (3055-3075) 5'-CGCCATTCTGTGGGTTCTAGA-3'	996
Forward ORF1-F7 (2990-3009) 5'-GACCCAAGGCAGATCCCTGC-3'†	
Reverse ORF1-R12 (4149-4171) 5'-GGCGGCCTCGAACTTCTCCTGAA-3'	1,182
Forward ORF1-F18 (3991-4011) 5'-ATTCACCACAGACGAGCCAGT-3'	
Reverse ORF2-R21 (5079-5100) 5'-GGTGATAGCCAATTGGTAAGCT-3'	1,110
Forward F13 (4896-4915) 5'-AATAACACTCTGGGCTGTAG-3'	
ReverseTX30SXN 5'-GACTAGTTCTAGATCGCGAGCGGCCGCCCTTTTTTTTTTTTTTTTTTTTTTTTTTTTTT-3'	2,092
Forward primer Anchor-1: 5'-CCTCTGAAGGTTCCAGAATCGATAG-3'	
Reverse primer ORF1-R14 (276-296) 5'-TAGACCTAGGGTGCGCACCGA-3'	§
Forward primer Anchor-2: 5'-GAATCGATAGTGAATTCGTG-3'	
Reverse primer ORF1-R13 (200-220) 5'-AACACGCTGTACCGGATGCGA-3'	240

*HEV, hepatitis E virus; ORF, open reading frame. †Numbers in a parentheses show the positions of primers corresponding to the entire genome of rat HEV V-105.  ‡Primer designed based on rat HEV (GU345042). §The PCR product was not detected (reverse).

Because 901 nt of V-105, corresponding to nt 4108–5008 of the R63 genome, were already known (*14*), primers F13 and open reading frame (ORF) 1–R12 were designed. An ≈2,100-nt fragment of the C-terminus of the rat HEV V-105, nt 4923-poly (A) tail, was amplified with a pair of primers, F13 and TX30SXN, by the first RT-PCR. The ORF1 region was amplified with primers ORF1-F1 and ORF1-R12. Two fragments, 440 nt (nt 11–450) and 1,182 nt (nt 2990–4171), were amplified by nested PCR with 2 sets of primers, ORF1-F2/ORF1-R1 and ORF1-F7/ORF1-R12, respectively. On the basis of the nucleotide sequences of those amplified fragments, ORF1-F9, ORF1-F16, ORF1-R16, ORF1-F18, and ORF2-R21 were designed, and 3 fragments, 1,830 nt (nt 388–2217), 996 nt (nt 2080–3075), and 1,110 nt (nt 3991–5100), were amplified with 3 sets of primers, ORF1-F9/ORF1-R10, ORF1F16/ORF1-R16, and ORF1-F18/ORF2-R21, respectively.

To amplify the N-terminus nonstructural region of V-105, we synthesized cDNA with primer ORF1-R14, and a DNA anchor (P-CACGAATTCACTATCGATTCTGGAACCTTCAGAGG-NH_3_) was linked to the N-terminus of the cDNA by T4 RNA Ligase I (BioLabs, Tokyo, Japan). By using this anchor-cDNA as the template, the first and the nested PCRs were carried out with 2 sets of primers, anchor-1/ORF1-R14 and anchor-2/ORF1-R13, respectively.

The V-105 genome consisted of 6,927 nt plus a poly (A) tail of a still-undetermined length (GenBank accession no. JX120573). The genomic structure of V-105 was, from the N-terminus toward the C-terminus, the N 5′–untranslated region (UTR) at nt 1–10, ORF1at nt 11–4900, ORF3 at nt 4917–5225, ORF2 at nt 4928–6862, the 3′-UTR at nt 6863–6927, and the poly (A) tail starting at nt 6928. ORF2 and ORF3 encode 644 aa and 102 aa, respectively, as do R63 and R68. However, ORF1 of V-105 encodes 1,629 aa, which is 7 aa shorter than either R63 or R68. The V-105 genome possessed 2 aa insertions (Ser-Pro) between the aa residues 591 and 592 and 9 aa deletions (Ser-Pro-Pro-Gly-Pro-Pro-Pro-Ala-Gly) between aa residues 852 and 853, corresponding to those of R63. The 3′-UTR was 65 nt as were R63 and R68. Unlike R63 and R68, only 1 additional putative ORF, corresponding to ORF4 (nt residues 27–578), was found in V-105, suggesting that other putative ORFs, ORF5 and ORF6 found in R63 and R68, are not common in rat HEV.

When the V-105 genome was compared with reported HEV genomes, the V-105 genome shared identities of only 50.5% with avian HEV, 53.6% with rabbit HEV, 53.7%–54.0% with wild boar HEV, and 53.1%–53.5% with HEV genotypes 1–4. In contrast, V-105 shared relatively high nucleotide sequence identities (76.8%–76.9%) with rat HEV strains (R63 and R68) (Table 2). The nucleotide and amino acid sequences of ORF1, ORF2, and ORF3 of V-105 were compared with those of other HEV genotypes, and the identities among them are shown in Table 2. Together, these results suggest that V-105 is more similar to rat HEV than to other HEV genotypes.

**Table 2:** Nucleotide and deduced amino acid sequence identities between human, wild boar, rabbit, rat, and avian HEV strains, compared with Vietnam rat HEV V-105 strain*

HEV strain (GenBank accession no.)	Entire genome		Vietnam rat HEV strain

Nucleotides, %				Amino acids, %		
ORF1	ORF2	ORF3	ORF1	ORF2	ORF3
Genotype 1 (NC_001434)	53.5		50.7	60.8	51.0		54.1	55.5	33.3
Genotype 2 (M74506)	53.3		51.2	59.1	51.4		53.1	54.6	30.6
Genotype 3 (AF060668)	53.3		50.8	59.2	53.8		51.6	57.1	26.5
Genotype 4 (AJ272108)	53.1		50.9	58.9	52.6		50.7	55.4	24.5
Wild boar HEV (AB573435)	53.7		51.5	59.6	53.4		50.3	56.3	28.2
Wild boar HEV (AB602441)	54.0		51.5	59.6	53.4		50.3	56.3	28.2
Rabbit HEV (FJ906895)	53.6		51.3	59.3	51.5		52.1	56.2	26.2
Rat HEV (GU345042)/R63	76.9		75.7	79.6	80.6		87.0	91.6	66.7
Rat HEV (GU345043)/R68	76.8		75.5	79.8	80.9		86.4	92.1	66.7
Avian (chicken) HEV (AY535004)	50.5		49.7	54.2	47.0		44.7	47.4	33.9

*HEV, hepatitis E virus; ORF, open reading frame.

Phylogenetic trees were generated on the basis of the nucleotide sequences derived from the entire genome and ORF3 of the genotypes 1–4, wild boar, rabbit, chicken, and rat HEV isolates. These trees demonstrated that V-105 does not belong to any known genotype and should probably be classified into a new genotype (Figure).

**Figure:**
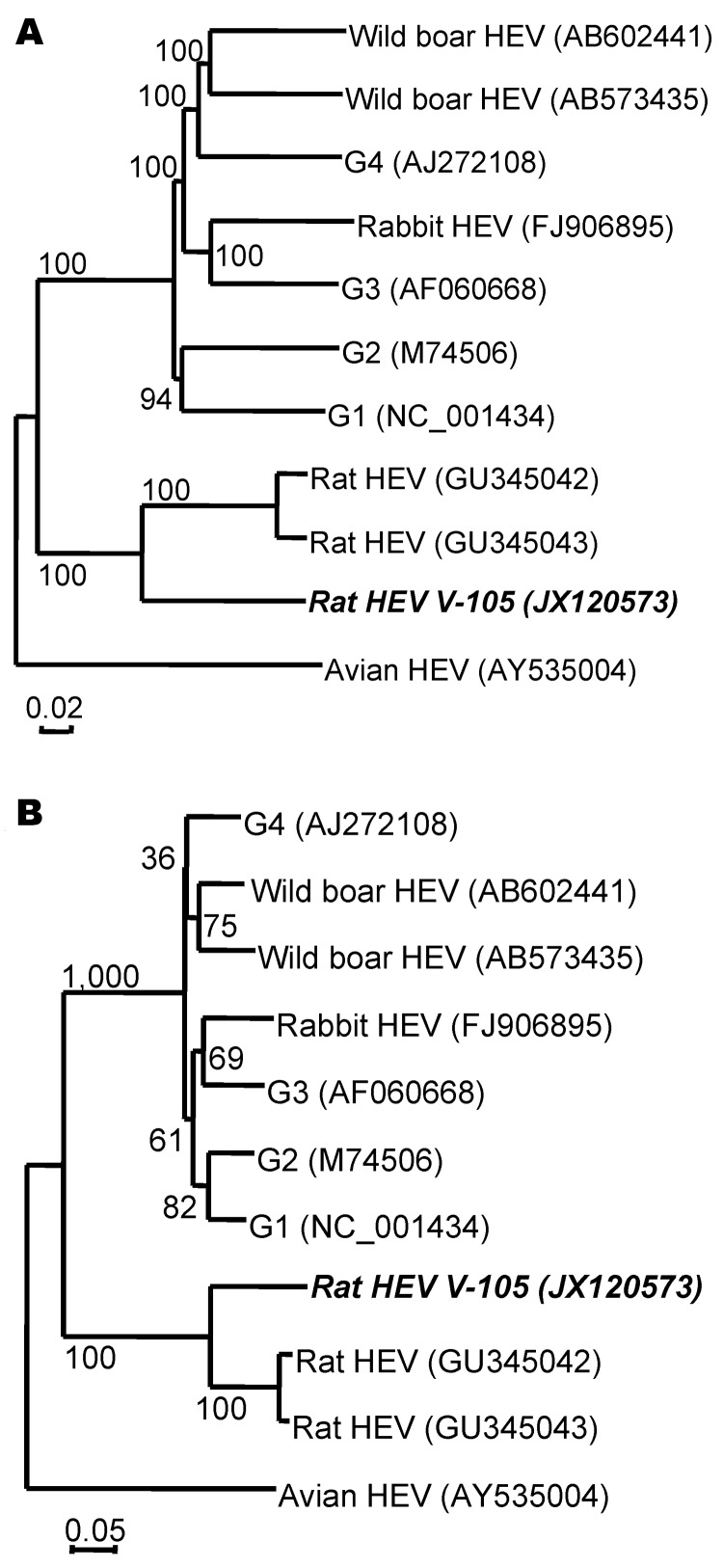
Phylogenetic relationships among genotypes 1–4, wild boar, rabbit, avian (bird), and rat hepatitis E virus (HEV) isolates. The nucleic acid sequence alignment was performed by using ClustalX 1.81 (www.clustal.org). The genetic distance was calculated by the Kimura 2-parameter method. A phylogenetic tree with 1,000 bootstrap replicates was generated by the neighbor-joining method, based on the entire genome (A) and open reading frame 3 (B) of the genotypes 1–4, wild boar, rabbit, chicken (avian), and rat HEV isolates. Scale bar indicates nucleotide substitutions per site. **Boldface **indicates isolates used in this study.

## Conclusions

In this study we successfully amplified the entire genome of an HEV strain isolated from a wild rat in Vietnam. Phylogenetic analyses and nucleotide and amino acid sequence comparisons demonstrated that the complete rat HEV genome sequences were consistently well separated from those of mammalian genotypes 1–4, wild boar, rabbit, and chicken HEV and close to those of the rat HEV strains. Although the entire genome of V-105 shared nucleotide sequence identities of only 76.8%–76.9% with the isolates from Germany (R63 and R68), the ORF1 and ORF3 amino acid identities between V-105 and these isolates were 86.4%–87.0% and 66.7%, respectively, which suggests that V-105 can be classified into a new genotype of rat HEV. However, ORF2 has relatively high amino acid identities with R63 and R68 (91.6%–92.1%), indicating that the V-105 and rat HEV isolates from Germany share similar antigenicity. In fact, rat HEV–like particles derived from R63 are cross-reactive to serum from V-105–infected wild rats (*14*).

In conclusion, we isolated and identified rat HEV strain V-105 from a wild rat in Vietnam, and this strain was highly divergent from known rat HEV isolates. We propose that the strain from Vietnam, V-105, is a new member of the rat HEV genotype.
